# Association of Neck Circumference with Anthropometric Indicators and Body Composition Measured by DXA in Young Spanish Adults

**DOI:** 10.3390/nu12020514

**Published:** 2020-02-18

**Authors:** María José Arias Téllez, Francisco M. Acosta, Guillermo Sanchez-Delgado, Borja Martinez-Tellez, Victoria Muñoz-Hernández, Wendy D. Martinez-Avila, Pontus Henriksson, Jonatan R. Ruiz

**Affiliations:** 1 Department of Nutrition, Faculty of Medicine, University of Chile, Independence 1027, Santiago 8380000, Chile; 2 PROFITH “Promoting Fitness and Health through Physical Activity” Research Group, Department of Physical and Sports Education, Sport and Health University Research Institute (iMUDS), Faculty of Sports Science, University of Granada, Ctra de Alfacar s/n, 18071 Granada, Spainborjammt@gmail.com (B.M.-T.); mariavmuher@gmail.com (V.M.-H.); wdma@hotmail.com (W.D.M.-A.);; 3 Pennington Biomedical Research Center, Baton Rouge, LA 70808, USA; 4 Department of Medicine, Division of Endocrinology, and Einthoven Laboratory for Experimental Vascular Medicine, Leiden University Medical Center, 2333 ZA Leiden, The Netherlands; 5 Department of Health, Medicine and Caring Sciences, Linköping University, 58183 Linköping, Sweden

**Keywords:** body fat distribution, cardiovascular risk, neck adipose tissue, obesity, upper body fatness

## Abstract

Background: Due to a clinical and public health interest of neck circumference (NC), a better understanding of this simple anthropometric measurement, as a valid marker of body composition is necessary. Methods: A total of 119 young healthy adults participated in this study. NC was measured over the thyroid cartilage and perpendicular to the longitudinal axis of the neck. Body weight, height, waist circumference (WC), and hip circumference were measured. A Dual X-ray absorptiometry (DXA) scan was used to determine fat mass, lean mass, and visceral adipose tissue (VAT). Additionally, body mass index (BMI) and triponderal mass index (TMI), the waist to hip and waist to height ratios, and the fat mass and lean mass indexes (FMI and LMI, respectively) were calculated. Results: NC was positively associated in women (W) and men (M), with BMI (rW = 0.70 and rM = 0.84, respectively), TMI (rW = 0.63 and rM = 0.80, respectively), WC (rW = 0.75 and rM = 0.86, respectively), VAT (rW = 0.74 and rM = 0.82, respectively), Waist/hip (rW = 0.51 and rM = 0.67, respectively), Waist/height (rW = 0.68 and rM = 0.83, respectively) and FMI (rW = 0.61 and rM = 0.81, respectively). The association between NC and indicators of body composition was however weaker than that observed by BMI, TMI, WC and Waist/height in both women and men. It is of note that in women, NC was associated with FMI, VAT and LMI independently of BMI. In men, adding NC to anthropometric variables did not improve the prediction of body composition, while slight improvements were observed in women. Conclusions: Taken together, the present study provides no indication for NC as a useful proxy of body composition parameters in young adults, yet future studies should explore its usefulness as a measure to use in combination with BMI, especially in women.

## 1. Introduction

Data from the European Health Interview Survey (Eurostat) indicates that more than half of the European population is overweight or obese [1]. Obesity virtually affects all ages and socioeconomic groups, and threatens to overwhelm both developed and developing countries [2]. Furthermore, obesity increases the risk of cardiovascular diseases and premature death [3].

Body weight and height are used to calculate body mass index (BMI) with the aim to classify individuals as underweight, normal weight, overweight, or as having obesity [4]. Nevertheless, BMI does not reflect body fat distribution [5,6]. Both waist circumference and waist-hip circumference ratio are indicators of body fat distribution, and they are strongly associated with cardiovascular disease [7,8]. However, these measures can be affected by the postprandial abdominal distension and breathing movement. In addition, people with or without obesity can have the same waist–hip ratio, making these measurements inappropriate to evaluate obesity [9].

Neck circumference has been proposed as an indicator of upper body fatness [10,11], as it has been associated with overweight and obesity phenotypes [12,13,14,15] as well as with cardiovascular disease risk factors [16,17,18,19,20]. Neck circumference is considered a practical measurement because, unlike other methods, it is easy to measure, it does not vary during the course of the day, it does not change with food intake or abdominal distension, it is not altered by inhalation or exhalation, and it can be measured without having to remove clothing [21,22]. The validity of neck circumference against reference methods such as computed axial tomography (TAC) and/or dual X-ray absorptiometry (DXA) has been studied in American [11,12,23,24], Canadian [25], Chinese [26], and English [27] individuals of both sexes, yet the results are limited to the association of neck circumference with total and abdominal body fat as well as with subcutaneous and visceral fat. However, whether neck circumference is associated with other parameters of body composition, such as lean mass, remains unknown.

Due to the clinical and public health interest about the utility of neck circumference, it is necessary to know if this measure is a valid marker of body composition. In addition, for a better understanding of the utility of neck circumference in the assessment of several chronic diseases in a healthy population, firstly it is necessary validate this anthropometric measure with body composition.

The aim of this study was to examine the association of neck circumference with indicators of anthropometry and body composition, including total and central body fat as well as lean body mass measured by DXA in young Spanish adults.

## 2. Materials and Methods

### 2.1. Participants

This cross-sectional study included a sample of 119 participants (82 women) aged 18 to 25 years old. The participants were enrolled in the ACTIBATE study (Clinical Trial Registration: NCT02365129 (ClinicalTrials.gov) [28], and were recruited through advertisements in electronic media and leaflets. All assessments were performed in Granada (south of Spain), during the months of October, November, and December 2016. The inclusion criteria were being healthy, not smoking or taking any medication, being sedentary (the participants reported to practice ˂20 min physical activity on ˂3 days/week), not having participated in a weight-loss program (body weight changes ˂3 kg over the last three months), and not having any cardiovascular disease. This study was approved by the Ethics Committee on Human Research of the University of Granada (n°924) and by the Servicio Andaluz de Salud (Centro de Granada, CEI-Granada) [28]. The study protocol and the written informed consent were performed in accordance with the Declaration of Helsinki (revision of 2013).

### 2.2. Neck Circumference Assessment

Neck circumference (cm) was measured using an inextensible metallic tape over the thyroid cartilage and perpendicular to the longitudinal axis of the neck [29]. During the measurement, the participant was in an anatomical position, standing or sitting with the head in the Frankfort plane and shoulders relaxed.

### 2.3. Anthropometric and Body Composition Measurements

Body weight (kg) and height (m) were measured using a calibrated digital scale SECA (model 769, Hamburg, Germany) and a portable stadiometer brand SECA (model 213) respectively. The participants wore light clothing and no shoes during the measurements. BMI (kg/m^2^) and Triponderal Mass Index (TMI, kg/m^3^) [30] were calculated. Waist circumference (WC) was measured in the minimum perimeter, at the end of a normal expiration, with the arms relaxed on both sides of the body. When the minimum perimeter could not be detected (such as in people who were overweight or had obesity), we took the measurements above the umbilicus, in a horizontal plane. Hip circumference was measured in the widest part of the gluteal region at the greater trochanter level [7]. We measured the perimeters of waist and hip (cm) twice with a plastic tape measure, and we used the average values for the analyses. We calculated the waist to hip ratio as well as the waist to height ratio.

In the same day in which the anthropometric measurements were performed, the participants underwent a Discovery Wi dual energy X-ray absorptiometry (Hologic, Bedford, MA, USA) scan in order to determine indicators of body composition, including fat mass, lean mass, and visceral adipose tissue (VAT). The participants underwent the scan with minimal clothing and not wearing any metal object. In addition, they were asked to stay as quiet and calm as possible during the scan time. Once the DXA scan was performed, we calculated the fat mass and lean mass indexes (FMI and LMI, respectively) [31] as fat or lean mass in kg divided by height in m^2^.

### 2.4. Statistical Analysis

The distribution of the variables was verified using the Shapiro–Wilk test, skewness and kurtosis values, visual check of histograms, Q-Q, and box plots. The descriptive parameters of women and men were compared with an independent sample t-test (equal variances) or with the Welch’s test (unequal variances).

Pearson correlations and multivariate stepwise forward linear regression analyses were used to examine (i) the association of neck circumference with anthropometric indicators (i.e., BMI, TMI, WC, W/hip, W/height) and body composition (i.e., FMI, LMI, VAT) and (ii) to examine the association of neck circumference and other anthropometric indicators (i.e., BMI, TMI, WC, W/hip, W/height) with body composition (i.e., FMI, LMI, VAT) starting from the one with highest simple correlation in the univariable analyses. Semipartial correlation was used as a measure of the relationship between FMI, VAT and LMI with independent variables of multivariate model, after controlling for the effect that one additional variable had on one of those variables. The level of significance was set at *p* < 0.05. The statistical analyses were performed using the Statistical Package for the Social Sciences (SPSS version 21.0, Chicago, IL, USA).

## 3. Results

**Figure 1:**
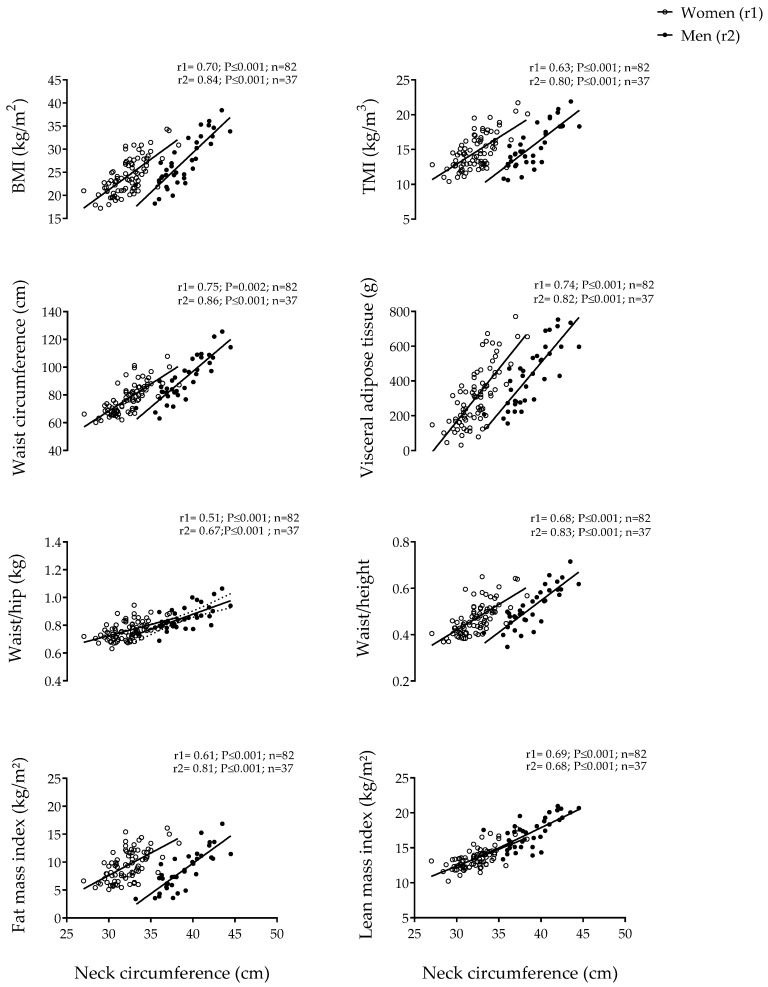
Association of neck circumference with indicators of anthropometry and body composition by sex (women: n = 82, men: n = 37). BMI: Body mass index; TMI: Triponderal mass index.

**Figure 2:**
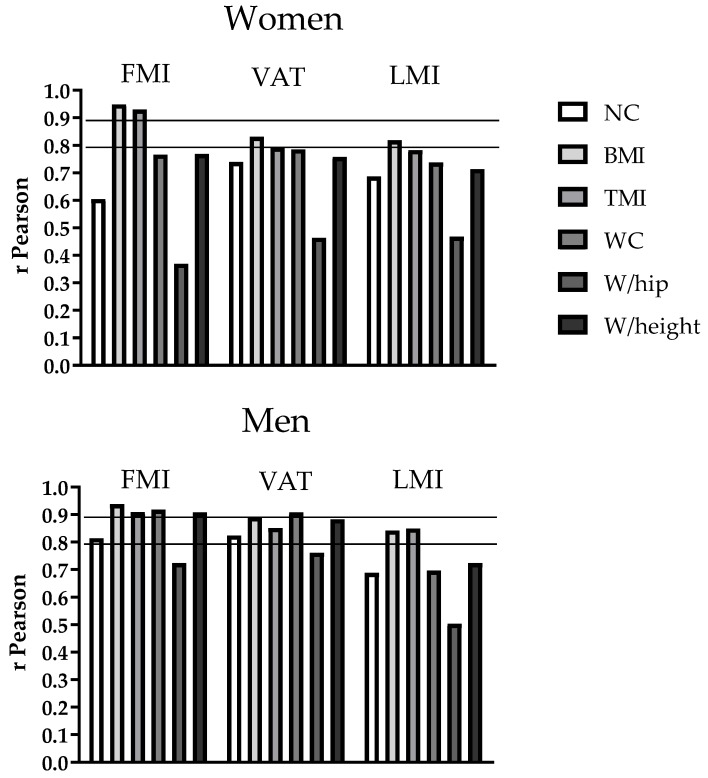
Association of anthropometric indicators with body composition measured by Dual X-ray absorptiometry (DXA) in women (n = 82) and men (n = 37). BMI: Body mass index; FMI: Fat mass index; LMI: Lean body mass index; NC: Neck circumference TMI: Triponderal mass index; VAT: Visceral adipose tissue; WC: Waist circumference; W/hip: Waist to hip ratio; W/height: Waist to height ratio.

The main characteristics of the study participants are presented in Table 1. Figure 1 shows the correlations of neck circumference with anthropometric indicators and body composition by sex. NC was significantly and positively associated in both women and men (all *p* ≤ 0.002)with BMI (rW = 0.70 and rM = 0.84, respectively), TMI (rW = 0.63 and rM = 0.80, respectively), WC (rW = 0.75 and rM = 0.86, respectively), VAT (rW = 0.74 and rM = 0.82, respectively), Waist/hip (rW = 0.51 and rM = 0.67, respectively), Waist/height (rW = 0.68 and rM = 0.83, respectively), FMI (rW = 0.61 and rM = 0.81, respectively), and LMI (rW = 0.69 and rM = 0.68, respectively). Figure 2 shows the correlations of NC and other anthropometric indicators with body composition measured by DXA in women and men. Pearson correlations of NC with indicators of body fat measured by DXA were consistently below 0.90. The association of neck circumference with indicators of body composition measured by DXA was weaker than that observed for BMI, TMI, WC and Waist/height, but not with Waist/hip. In women, BMI (univariate model) was the strongest predictor of FMI (R^2^ = 0.899, *p* ≤ 0.001), of VAT (R^2^ = 68.6, *p* ≤ 0.001) and of LMI (R^2^ = 66.7, *p* ≤ 0.001). Neck circumference was associated with FMI, VAT and LMI independently of BMI (Table 2, multivariate model). However, in men, BMI (univariate model) was the strongest predictor of FMI (R^2^ = 87.6, *p* ≤ 0.001) followed by WC (multivariate model) with a variance explained of 5%. In addition, WC (univariate model) was the strongest predictor of VAT (R^2^ = 82.0, *p* ≤ 0.001) followed by BMI. Finally, TMI was the unique predictor of LMI (R^2^ = 71.5, *p* ≤ 0.001).

## 4. Discussion

In the present study we showed that neck circumference is associated with anthropometric indicators including BMI, TMI, or WC as well as with indicators of body fat measured by DXA such as FMI or VAT in a sample of young Spanish adults. In addition, we observed a positive association between neck circumference and LMI. It is of note that these associations appeared to be stronger in men than in women. The correlation of neck circumference with indicators of body composition measured by DXA was lower than that observed for other classic anthropometric indicators such as BMI and WC, which limits the value of neck circumference as a useful proxy of body composition parameters in young adults. However, in women, neck circumference was associated with all three measures of body composition independently of BMI and, therefore, it might be worth exploring in future studies its usefulness as a measure to use in combination with BMI.

Several studies examined the association between neck circumference and indicators of body composition in adults from different ethnic groups or races [11,12,23,24,25,26,27] of both sexes. Castro-Pinero et al. [32] showed weaker associations between neck circumference and FMI in girls 191 (r = 0.494, *p* < 0.001) and in boys (r = 0.474, *p* < 0.001) than in our study, most likely due to the fact that they estimated FMI from skin-fold thickness. Studies utilizing computer tomography as the reference method to asses body composition showed a positive and significant association of neck circumference with VAT and subcutaneous adipose tissue [11,23,26], whereas others only found a significant association with VAT [12,27]. On the other hand, studies assessing body composition by DXA [24,25] have shown that neck circumference is associated with the percentage of total body fat and abdominal fatness. Similarly, our findings showed that neck circumference was positively associated with FMI and VAT estimated by DXA. Thus, it seems that neck circumference is a valid marker of total and central body fat in young adults, and that it could be implemented as an easy and practical measure. Interestingly, we observed that neck circumference was highly correlated to LMI, which was independent of BMI in women but not in men. To our knowledge, there are no studies investigating the association of neck circumference with LMI, which hamper between-studies comparisons.

Regarding anthropometric indicators, Ben-Noun et al. [22] showed, for the first time, that neck circumference was positively associated with BMI (women, r = 0.71; men, r = 0.83), WC (women, r = 0.85; men, r = 0.86), hip circumference (women, r = 0.56; men, r = 0.62), and waist/hip ratio (women, r = 0.87; men, r = 0.66) in adults. Later studies found similar results in Turkish [33], Pakistani [13], and Chinese [18,34] populations. In agreement with these studies, we observed that neck circumference was positively associated with BMI and TMI, and with anthropometric measures related to body fat distribution (i.e., WC, Waist/hip and Waist/height) in a sample of young Spanish adults.

Although neck circumference is an anthropometric indicator at least as simple as BMI and easier than WC in patients with weight excess, it might not add new information on body composition compared with other classic anthropometric indicators. We observed that the association of neck circumference with FMI, VAT, and LMI was weaker than that observed for BMI, TMI, WC and WC/height. In women, NC slightly improved the prediction of LMI, VAT and FMI beyond BMI. Future studies should explore its usefulness as a measure to use in combination with BMI. Assyov et al. [35] showed that WC was the best anthropometric measure to predict the distribution of adipose tissue measured by means of Body Impedance Analyse (BIA) in men and women with obesity (45–70 years old). Similar results were found by Joshipura et al. [36] in overweight or obese individuals (40–65 years old), showing that BMI and WC were better correlated with body fat percentage (BIA) than neck circumference. It is however relevant that neck circumference seems to be more strongly associated with cardiovascular disease risk factors than other anthropometric indicators such as BMI or WC [35,36]. Consequently, although the available evidence points out that neck circumference might not be the best marker of body composition, its role as a predictive and easy tool to assess other cardiovascular disease risk factors should be further considered.

The cross-sectional nature of this study prevents us from determining any causality in the results. Our results are limited to young adults, and, therefore, whether neck circumference is a valid marker of body composition in older adults and people with cardio metabolic disease are not known. Furthermore, although DXA is a valid and extensively used method to assess body composition, further studies should consider the use of reference methods such as computed axial tomography or magnetic resonance imaging. In addition, our findings are limited by the sample size, and the differences of strength of the association between women and men could be driven for the differences of body composition and not for the sex. The present study is exploratory, without external validation.

## 5. Conclusions

In conclusion, neck circumference is associated with anthropometric indicators such as BMI and WC as well as with indicators of body composition measured by DXA (FMI, VAT, LMI), but the results indicate that it is not a better predictor of total and central body fat than other classic anthropometric markers as BMI or WC in young healthy adults. Taken together, the present study provides no indication for neck circumference as a useful proxy of body composition parameters in young adults, yet future studies should explore its usefulness as a measure to use in combination with BMI, especially in women.

## Figures and Tables

**Table 1:** Characteristics of the participants.

	All (n = 119)	Women (n = 82)	Men (n = 37)	*p*
Age (years)	21.9 (2.3)	21.8 (2.2)	22.1 (2.4)	0.488
Weight (kg)	71.7 (16.4)	66.0 (11.6)	84.9 (18.0)	<0.001
Height (m)	1.69 (8.5)	1.65 (6.5)	1.77 (6.3)	<0.001
Neck circumference (cm)	34.3 (3.8)	32.3 (2.1)	38.8 (2.6)	<0.001
BMI (kg/m^2^)	25.1 (4.6)	24.1 (4.0)	27.2 (5.3)	0.003
TMI (kg/m^3^)	14.9 (2.7)	14.7 (2.5)	15.4 (3.0)	0.193
WC (cm)	81.6 (13.8)	77.4 (11)	90.1 (15.3)	<0.001
Waist/hip	0.85 (0.1)	0.80 (0.1)	0.85 (0.1)	<0.001
Waist/height	0.48 (0.08)	0.47 (0.07)	0.52 (0.09)	0.006
Fat mass (kg)	26.0 (8.8)	26.0 (7.5)	27.0 (11.3)	0.594
FMI (kg/m^2^)	9.1 (3.0)	9.4 (2.7)	8.5 (3.5)	0.124
VAT (g)	348.5 (181.8)	307.4 (168.0)	439.4 (181.0)	<0.001
Lean mass (kg)	42.0 (9.9)	37.0 (5.0)	53.7 (7.6)	<0.001
LMI (kg/m^2^)	14.6 (2.4)	13.5 (1.5)	17.2 (2.2)	<0.001

Values are means ± standard deviation. *p* for sex comparisons. BMI: Body mass index; FMI: Fat mass index; LMI: Lean mass index; TMI: Triponderal mass index; VAT: Visceral adipose tissue; WC: Waist circumference.

**Table 2:** Association of neck circumference, body mass index, triponderal mass index, waist circumference, waist to hip ratio and waist to height ratio with body composition measured by Dual X-ray absorptiometry (DXA) in women (n = 82) and men (n = 37).

WOMEN												
	FMI				VAT				LMI			
	β	95% CI	R^2^	sr	β	95% CI	R^2^	sr	β	95% CI	R^2^	sr
Univariable model												
- NC	3.765 (0.554) ***	2.662 to 4.867	0.358		282.857 (28.706) ***	225.731 to 339.983	0.543		2.277 (0.270) ***	1.741 to 2.813	0.465	
- BMI	3.025 (0.112) ***	2.801 to 3.248	0.899		162.546 (12.172) ***	138.323 to 186.769	0.686		1.391 (0.109) ***	1.175 to 1.608	0.667	
- TMI	2.708 (0.120) ***	2.470 to 2.946	0.863		141.522 (12.208) ***	117.227 to 165.817	0.622		1.215 (0.108) ***	1.000 to 1.429	0.608	
- WC	2.696 (0.253) ***	2.193 to 3.199	0.582		169.522 (814.961) ***	139.749 to 199.295	0.611		1.384 (0.141) ***	1.103 to 1.665	0.539	
- W/hip	1.281 (0.360) ***	0.565 to 1.997	0.126		98.527 (21.049) ***	56.638 to 140.416	0.205		0.864 (0.182) ***	0.502 to 1.227	0.210	
- W/height	2.360 (0.220) ***	1.923 to 2.797	0.586		142.782 (13.746) ***	115.427 to 170.137	0.569		1.168 (0.128) ***	0.913 to 1.422	0.504	
Multivariable model												
- BMI - NC	3.269 (0.153) *** −0.684 (0.298) *	2.965 to 3.573 −1.277 to −0.090	0.904	0.735 −0.079	119.758 (15.622) *** 119.875 (30.504) ***	88.664 to 150.853 59.159 to 180.592	0.734	0.439 0.225	1.125 (0.147) *** 0.746 (0.286) *	0.833 to 1.417 0.176 to 1.316	0.690	0.475 0.161
**MEN**												
Univariable model											
- NC	5.115 (0.616) ***	3.864 to 6.367	0.653		270.139 (31.414) ***	206.366 to 333.912	0.670		2.717 (0.490) ***	1.723 to 3.710	0.453	
- BMI	2.800 (0.175) ***	2.444 to 3.156	0.876		138.745 (12.002) ***	114.380 to 163.109	0.787		1.590 (0.172) ***	1.241 to 1.939	0.701	
- TMI	2.775 (0.214) ***	2.340 to 3.211	0.822		135.489 (14.154) ***	106.754 to 164.224	0.716		1.640 (0.172) ***	1.292 to 1.988	0.715	
- WC	2.839 (0.207) ***	2.420 to 3.259	0.839		146.521 (11.426) ***	123.325 to 169.717	0.820		1.360 (0.237) ***	0.879 to 1.842	0.470	
- W/hip	2.475 (0.398) ***	1.667 to 3.284	0.511		135.740 (19.555) ***	96.040 to 175.439	0.567		1.086 (0.316) **	0.445 to 1.727	0.231	
- W/height	2.777 (0.217) ***	2.337 to 3.217	0.819		140.969 (12.658) ***	115.273 to 166.666	0.774		1.399 (0.226) ***	0.941 to 1.857	0.510	
Multivariable model												
- BMI - WC - TMI	1.788 (0.400) *** 1.144 (0.414) **	0.975 to 2.601 0.303 to 1.986	0.896	0.241 0.149	56.675 (26.110) * 92.791 (27.033) **	3.612 to 109.738 37.855 to 147.728	0.837	0.146 0.231	1.640 (0.172) ***	1.292 to 1.988	0.715	0.850

Multivariate stepwise regression analysis to examine the association of anthropometric indicators with FMI, VAT and LMI. All the independent variables were standardized (Z-score). β coefficient (standard deviation), 95% confidence interval (CI), adjusted coefficient of determination (R2), sr (semipartial correlation) and *p*-value are provided. * *p* ≤ 0.05, ** *p* ≤ 0.01, *** *p* ≤ 0.001. BMI: Body mass index; FMI: Fat mass index; LMI: Lean body mass index; NC: Neck circumference; TMI: Triponderal mass index; VAT: Visceral adipose tissue; WC: Waist circumference; W/hip: Waist to hip ratio; W/height: Waist to height ratio.
